# Endolymphatic Hydrops in Fluctuating Hearing Loss and Recurrent Vertigo

**DOI:** 10.3389/fsurg.2021.673847

**Published:** 2021-05-31

**Authors:** Pablo Domínguez, Raquel Manrique-Huarte, Víctor Suárez-Vega, Nieves López-Laguna, Carlos Guajardo, Nicolás Pérez-Fernández

**Affiliations:** ^1^Department of Radiology, Clínica Universidad de Navarra, Pamplona, Spain; ^2^Navarra Institute for Health Research (IdiSNA), Pamplona, Spain; ^3^Department of Otorhinolaryngology, Clínica Universidad de Navarra, Pamplona, Spain; ^4^Department of Radiology, Clínica Universidad de Navarra, Madrid, Spain; ^5^Department of Emergency Medicine, Clínica Universidad de Navarra, Madrid, Spain; ^6^Escuela de Fonoaudiología, Universidad Austral de Chile, Sede Puerto Montt, Valdivia, Chile; ^7^Department of Otorhinolaryngology, Clínica Universidad de Navarra, Madrid, Spain

**Keywords:** Ménière's disease, endolymphatic hydrops, MRI, fluctuating hearing loss, recurrent vertigo, vestibular tests

## Abstract

**Background:** Endolymphatic hydrops (EH) is the histopathological hallmark of Ménière's disease (MD) and has been found by *in vivo* magnetic resonance imaging (MRI) in patients with several inner ear syndromes without definite MD criteria. The incidence and relevance of this finding is under debate.

**Purpose:** The purpose of the study is to evaluate the prevalence and characteristics of EH and audiovestibular test results in groups of patients with fluctuating audiovestibular symptoms not fulfilling the actual criteria for definite MD and compare them with a similar group of patients with definite MD and a group of patients with recent idiopathic sudden neurosensory hearing loss (ISSNHL).

**Material and Methods:** 170 patients were included, 83 with definite MD, 38 with fluctuating sensorineural hearing loss, 34 with recurrent vertigo, and 15 with ISSNHL. The clinical variables, audiovestibular tests, and EH were evaluated and compared. Logistic proportional hazard models were used to obtain the odds ratio for hydrops development, including a multivariable adjusted model for potential confounders.

**Results:** No statistical differences between groups were found regarding disease duration, episodes, Tumarkin spells, migraine, vascular risk factors, or vestibular tests; only hearing loss showed differences. Regarding EH, we found significant differences between groups, with odds ratio (OR) for EH presence in definite MD group vs. all other patients of 11.43 (4.5–29.02; *p* < 0.001). If the ISSNHL group was used as reference, OR was 55.2 (11.9–253.9; *p* < 0.001) for the definite MD group, 9.9 (2.1–38.9; *p* = 0.003) for the recurrent vertigo group, and 5.1 (1.2–21.7; *p* = 0.03) for the group with fluctuating sensorineural hearing loss.

**Conclusion:** The percentage of patients with EH varies between groups. It is minimal in the ISSNHL group and increases in groups with increasing fluctuating audiovestibular symptoms, with a rate of severe EH similar to the known rate of progression to definite MD in those groups, suggesting that presence of EH by MRI could be related to the risk of progression to definite MD. Thus, EH imaging in these patients is recommended.

## Introduction

In recent years, the diagnostic approach to patients with Ménière's disease (MD) has undergone a major challenge. Two almost simultaneous facts explain this situation. First, the unconditional acceptance of new guidelines and criteria for the clinical diagnosis of this disease and, second, the appearance of methods for the *in vivo* visualization of endolymphatic hydrops (EH). Each aims to improve the management of patients with recurrent non-positional vertigo, but the former ignores the contribution of the latter, which, in turn, critically demands a framework of diagnosis defining a clinical perspective on the new findings in magnetic resonance imaging (MRI) of the inner ear.

The main differences between the published diagnostic criteria of MD have been critically reviewed by previous authors and show that some are extremely restrictive to a complex symptom presentation or, on the contrary, permit partial manifestations that resemble the main fluctuating nature of symptoms in the disease (1). As MD is part of a wider scenario of fluctuating hearing loss or recurrent vertigo, most of the guidelines do not consider some forms of staging or of status, both of which influence vestibular function (2) and auditory tests results (3). Another generalized deficiency in the guidelines is the absence of complete oto-neurological examinations to further categorize the level of vestibular deficit. The need for a complete otoneurologic evaluation of patients with suspected MD, at their first presentation or during follow-up, is the main conclusion of several studies that addressed particular clinical characteristics. A detailed scrutiny of symptoms has shown the existence of different subtypes or clinical variants in the unilateral (4) and bilateral (5) presentations of definite MD. This has also occurred in the case of bedside vestibular examination (6), auditory (7), and vestibular testing (8, 9).

MRI detection of EH is the result of high tech combined with complex sequence parametrization to allow the identification of the minute sensory and supporting structures of the inner ear within very hard dense bone. The imaging basis of EH relies on the fact that gadolinium-based MR contrast diffuses to the perilymph but not to the endolymph, altering the perilymph signal and allowing later discrimination between both components in MR imaging (10). At present, two sequences: “Fluid attenuated inversion recovery” (FLAIR) and “Inversion Recovery with REAL reconstruction” (REAL-IR), and two methods of contrast administration (intravenous or intratympanic) have been consolidated. This creates a source of variability that may influence the results obtained, but the technique is now widely accepted. Excellent reviews of the available techniques have also been published (11, 12). The scarce, but otherwise illustrative, number of otopathology reports on patients with any inner ear disorder representative of, or clinically ascribed to, MD is a magnificent platform for comparing results (13). It has demonstrated the importance of EH in the natural history of MD (14).

In this work, we address the EH MRI findings of patients with fluctuating auditory and vestibular symptoms, both in isolation and in combination. This is, first, a descriptive work that aims to clarify the rationale for a more in-depth evaluation of patients with fluctuating inner ear symptoms. The second purpose for this study comes from the well-known association between recurrent vestibulopathy (RV) and MD (15), as well as between fluctuating sensorineural hearing loss (FSNHL) and MD (16). In both cases, MD is expected to develop in 4% and 10–37% of the cases followed, respectively. This is the percentage of patients with EH we expect to find with MRI evaluation.

## Materials and Methods

### Patients

The patients included in this work were seen at two different venues by experienced otologists–neurotologists (RMH, NPF), and all the tests included were performed for their routine care and evaluation. All data shown were retrospectively obtained from their digital medical history at both institutions. All research has been conducted in accordance with the World Medical Association's Declaration of Helsinki and in accordance with institutional and national guidelines. Patients gave written authorization for the use of their medical data for research purposes. No identifiable human data are shown.

For this study the following patients were included (Table 1):

1. Patients with unilateral “definite” MD according to the guidelines defined by the American Academy of Otolaryngology Head and Neck Surgery in 1995 (17), who also fulfilled the criteria of “definite” MD according to Barany 2015 (18).
2. Patients with so-called “atypical” MD. We have used a previously defined categorization that is based on the main fluctuating symptom and provides five additional groups (19). In the case of fluctuating sensorineural hearing loss (FSNHL), this can occur with a single attack of vertigo alone, with concurrent unsteadiness or without any vestibular symptoms. In the case of recurrent vertigo (RV), it may occur with fixed sensorineural hearing loss or without hearing loss (19).
3. Patients with unilateral idiopathic sudden sensorineural hearing loss (ISSNHL) without vertigo or dizziness (20).

**Table 1:** Definitions for inclusion in this study.

**Term**	**Original definition**	**References**
MD definite	•Two or more definitive spontaneous episodes of vertigo lasting 20 min or longer •Audiometrically documented hearing loss on at least one occasion •Tinnitus or aural fullness in the affected ear •Other causes excluded	(17, 18)
Atypical MD cochlear (FSNHL group)	Patients with fluctuating hearing loss •Single vertigo type: with a single episode of vertigo •Unsteady type: without vertigo but with unsteadiness •No vestibular symptom type: entirely without vestibular symptoms	(19)
Atypical MD vestibular (RV group)	Patients with recurrent episodic vertigo •Hearing loss type: with complicating fixed sensorineural hearing loss •Normal hearing type: with normal hearing	(19)
Idiopathic sudden sensorineural hearing loss (ISSNHL group)	•Sensorineural hearing loss >30 dB HL, in three or more consecutive frequencies, over <72 h	(20)

*MD, Ménière's disease; FSNHL, fluctuating sensorineural hearing loss; RV, recurrent vertigo; ISSNHL, idiopathic sudden sensorineural hearing loss; dB HL, decibels hearing level*.

Exclusion criteria were middle ear disease, previous surgical or intratympanic treatment and tumors, or vascular compression in the vestibular nerve.

### Procedures

#### Clinical Data and Audiovestibular Tests

After clinical evaluation and bedside vestibular examination, tests of the vestibulo-ocular reflex (video head-impulse test or vHIT, and ocular vestibular-evoked myogenic potential or oVEMP), of the vestibulo-spinal reflex (cervical vestibular-evoked myogenic potential, or cVEMP), of nystagmus (caloric test), and of hearing (pure tone audiometry) were performed. After signing the informed consent, the MRI study was performed.

##### Clinical Evaluation and Bedside Testing

Data recorded for all patients included “disease duration” defined as time in years since the first typical episode of vertigo or hearing fluctuation or, in the case of ISSNHL, in days since it began as described by the patient. For fluctuating symptoms, its severity was evaluated according to the number of episodes (vertigo or hearing fluctuation) in the previous 6 months for inclusion in the study and its activity by the time (days) since the last vertigo crisis or hearing fluctuation. Additionally, the existence of drop attacks of the Tumarkin type was also considered in the case of patients with recurrent vertigo. All patients were evaluated for the existence of migraine and vascular risk factors.

Patients underwent a complete neurotological examination, and particular attention was paid to the appearance of spontaneous nystagmus behind Frenzel goggles. In addition, horizontal post head-shaking nystagmus or vibration-induced nystagmus and positional nystagmus were considered.

##### Audiometric Test

Audiometric findings are reported in terms of the 0.25- to 6-kHz pure-tone thresholds expressed in decibel hearing level (dB HL). In order to better analyze the results, the audiometry was divided into (a) low-frequency range threshold (0.25 kHz), (b) mean pure-tone average (PTA) for the 0.5, 1, 2, and 3 kHz, and (c) high-frequency range mean threshold (4 and 6 kHz). The affected and non-affected ears are then analyzed. For each range of frequencies, we analyze the number of patients who display a threshold lower than 50 dB HL vs. those equal or higher than 50 dB HL (21).

##### Vestibular Function Tests

The results of the video head impulse test (vHIT: GN Otometrics, Denmark) will be given as the gain of the vestibulo-ocular reflex and the appearance of refixation saccades obtained for head impulses in the plane of each of the three semicircular canals (SCC) of the affected and unaffected ear. The mean gain is considered normal for each of the canals in each patient evaluated when above the lower limit for the patient's age as given in the system used. In the case where the gain is found to be lower than expected, it will be considered abnormal if there are refixation saccades (overt or covert). A test is considered normal when all three SCCs are normal and abnormal when at least one of the SCCs is abnormal.

The caloric test will be considered abnormal if canal paresis (in accordance with Jongkees' formula) is above 22%.

For vestibular-evoked myogenic potential (VEMP) testing, both cervical (cVEMP) and ocular (oVEMP) tests, the normal vestibular function is defined as the presence of vestibular-evoked myogenic potentials in both ears. It is analyzed by the inter-aural asymmetry ratio [IAAR (%)] for air-conducted stimulation at 0.5 kHz. The intensity of the acoustic stimulus used is 97 dB normalized HL. A Blackman envelope was configured (rise/fall time 2 ms, plateau time 0 ms). One hundred averages were presented at a rate of 5.1/s. The cVEMP is recorded with the patients seated in an upright position. The signals obtained were rectified by the contraction value of the ECM muscle. The oVEMP is recorded with the patient sitting upright, having been instructed to look at a fixed point on the wall with an upward inclination of 35°.

Abnormal vestibular function is defined as either a unilaterally or bilaterally absent function. An absent oVEMP response is defined as EMG recordings lacking definable n10 waves, and an absent cVEMP response is defined as EMG recordings lacking definable p13 waves. The number of recordings made per subject is based on the reproducibility of the observed response. In cases in which the response is considered absent, the mean amplitude will be null (0 μV). To calculate the IAAR, the mean null values were artificially set at 1 μV, as was described in the work of Jerin et al. (22). In the case of recognized waves after stimulation of both ears, the upper limit of normal IAAR, 30%, was the criteria to define a normal or abnormal test in our locale (23).

#### Magnetic Resonance Imaging Endolymphatic Hydrops Evaluation

In all patients, exclusion of contraindications for MR imaging and/or intravenous contrast administration was checked prior to contrast administration. MR studies were performed in two 3T MR machines, either a Siemens Magnetom Vida (Siemens Healthineers, Erlangen, Germany) with a dedicated Siemens 20-channel head-coil, or a Siemens Magnetom Skyra with a dedicated Siemens 32-channel head coil. For this study, a REAL-IR sequence based on the previous publication by Naganawa et al. was performed (24). This was done on all patients, 4–5 h after the intravenous administration of a single dose of paramagnetic contrast material (0.1 ml/kg of Gadovist 1M, Bayer AG, Berlin, Germany) via an antecubital vein. Heavily T2-weighted cisternography images were also obtained in all patients for anatomical assessment.

MRI studies were evaluated by one of two different neuroradiologists, both with years of experience in EH imaging evaluation. Dubious cases were resolved in consensus. Only one patient was excluded due to insufficient quality of imaging.

**Figure 1:**
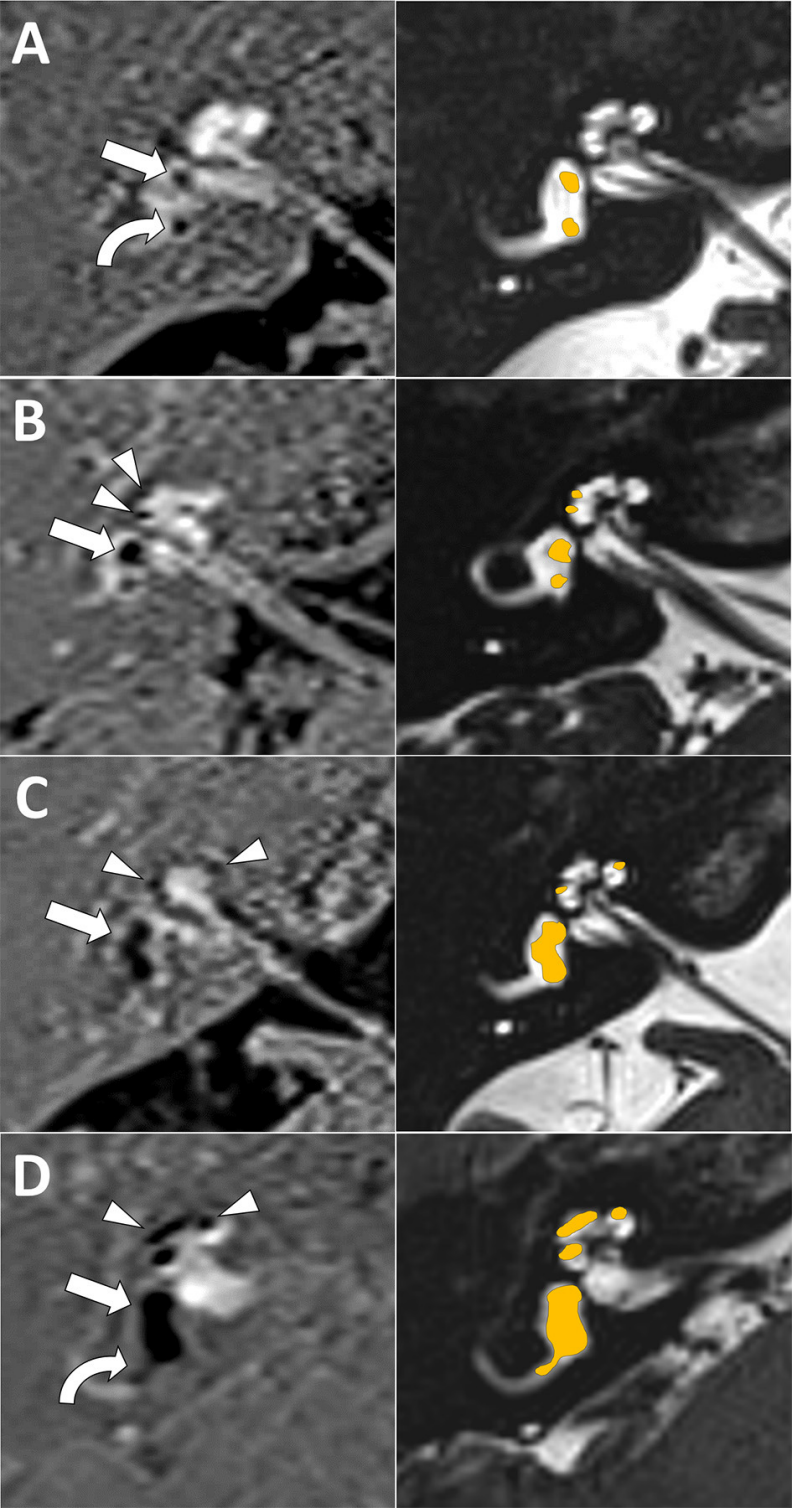
Magnetic resonance imaging (MRI) endolymphatic hydrops (EH) visual scale examples. First column is inversion recovery with real reconstruction (REAL-IR) images for EH evaluation, second column is corresponding anatomical images with superimposed schematic colored EH as reference. **(A)** Grade 0 (normal, no EH) cochlear and vestibular EH (only normal saccule, straight arrow, and utricle, curved arrow, are seen). **(B)** Grade 1 cochlear (mild EH, arrow heads) and grade 1 vestibular EH (mild EH, with dilated saccule larger than utricle but not fused, arrow) is seen. **(C)** Grade 2 vestibular EH (moderate EH, with saccule and utricle dilated and fused, arrow); note also grade 1 cochlear EH (arrow heads). **(D)** Grade 2 cochlear (severe EH, arrow heads) and grade 3 vestibular EH (severe EH, saccule and utricle occupying almost all the vestibule, straight arrow); note also herniation toward the non-ampullary end of the horizontal semicircular canal (curved arrow).

Cochlear EH was visually evaluated with a three-grade visual scale (none, mild, or severe) in accordance with previous work (25). Briefly, cochlear EH is evaluated at the axial slice closer to the modiolus and better depicting all cochlear turns (midmodiolar plane). In grade 0 (none), Reissner's membrane is not displaced, in grade 1 (mild), Reissner's membrane is displaced, but the scala media (cochlear duct) occupies less than half the scala vestibuli, and in grade 2 (severe), it occupies half or more of the scala vestibuli (Figure 1).

Vestibular EH was also visually evaluated but with a four-grade scale as previously published (12, 26). Briefly, vestibular EH is evaluated at the axial slice better depicting the horizontal semicircular canal (HSC). Grade 0 (none) corresponds to normal saccule and utricle, grade 1 (mild) corresponds to saccule dilatation but without fusion with the utricle, grade 2 (moderate) corresponds to dilated and fused saccule and utricle but with a clear rim of peripheric perilymph, and in grade 3 (severe), almost all the vestibules are occupied by endolymph (Figure 1).

The presence or absence of vestibular EH herniation toward the non-ampullar end of the HSC (27) and presence or absence of asymmetric perilymph hyperintensity were also recorded (28, 29).

### Statistics

Quantitative variables are presented as mean values and standard deviation (SD), and categorical variables are presented as percentages (%).

For the comparison of baseline characteristics of the patients, the four groups according to Kimura's classification were used (FSNHL, RV, MD definite, and ISSNHL). The same distribution of patients was used for the comparison of audiometric thresholds below or above 50 dB HL, and for the findings in the vestibular examination. Parametric and non-parametric tests were used for comparison, as appropriate.

Logistic proportional hazard models were used to obtain the odds ratios (OR) for hydrops presence between groups. For this analysis, we first grouped participants into two categories: “definite MD” vs. “atypical MD + ISSNHL” (all other patients), considering the “atypical MD + ISSNHL” as the reference category. Afterward, we repeated the analysis using the ISSNHL group as the reference category and compared it with the other three groups: definite MD, FSNHL (merging its three diagnostic subcategories; no vestibular symptoms, unsteadiness, and vertigo), and RV (merging its two subcategories). We fitted an age- and sex-adjusted model and a multivariable adjusted model for potential confounders. The adjusted model included as covariates age, sex, vascular risk factors, hypertension, dyslipidemia, and diabetes.

A *p*-value below 0.05 was considered statistically significant. Analyses were performed with STATA version 13.0.

## Results

We have included 170 patients of which 90 were female (52.9%) and 80 were male (47.1%). At diagnosis, the mean age was 54 ± 14 years. The right ear was affected in 78 patients, while the left was affected in 92. According to the classification used, the main patient characteristics in each group are shown in Table 2. Of the 170 patients included, 83 had definite MD, 38 had FSNHL (15 without vestibular symptoms, 15 with unsteadiness, and eight with vertigo), 34 had RV (nine without SNHL and 25 with fixed SNHL), and 15 had ISSNHL. As seen from the data, the groups were homogeneous in almost all the characteristics evaluated. No statistically significant differences regarding disease duration, episodes, Tumarkin spells, migraine, or vascular risk factors are depicted. As shown in the table, for the variable “disease duration,” the ISSNHL group was not included in the analysis as it was measured in days, while all others were measured in years, nor was this group included in the analysis for two other non-applicable variables: number of episodes and Tumarkin crises.

**Table 2:** Baseline characteristics of the patients.

**Group**		**FSNHL**			**RV**		**MD definite**	**ISSNHL**	***p***
**Subgroup**		**No vestibular symptoms**	**Unsteadiness**	**Vertigo**	**No HL**	**Plus HL**			
*N*		15	15	8	9	25	83	15	
Age, years (SD)		50.0 (14.6)	54.7 (14.5)	46.3 (22.4)	47.9 (12.8)	57.0 (16.3)	55.1 (11.9)	58.1 (7.5)	0.30
Sex (women) (%)		60	60	75	55.6	48	50.6	46.7	0.82
Side, right (%)		26.7	60.0	50.0	55.6	60.0	40.9	46.7	0.35
Disease duration, years (SD)		3.6 (4.8)	4.2 (6.7)	1.4 (1.2)	5.0 (9.8)	5.2 (6.7)	5.3 (6.2)	–	0.18
Days since last vertigo spell or hearing loss (% of the total patients in each category)	<30	6.3	6.3	5.4	4.5	11.7	55.3	13.5	0.02*
	≥30	2.2	15.6	2.2	8.9	22.2	48.9	0	
Episodes (%)	<10	100	85.7	100	88.9	73.9	79.3	–	0.59
	10–20	0	7.1	0	11.1	21.7	17.1	–	
	≥20	0	7.1	0	0	4.4	3.7	–	
Tumarkin, positive (%)		7.7	0	0	0	8.7	15.7	–	0.23
Migraine (%)	No headache	93.3	93.3	87.5	77.8	91.7	86.6	66.6	0.26
	Migraine	0	6.7	12.5	11.1	8.3	10.9	11.1	
	Tensional	6.7	0	0	11.1	0	2.4	22.2	
Vascular risk factors, presence (%)		6.7	33.3	25.0	22.2	28.0	28.1	6.7	0.36

N, number of patients; SD, standard deviation; FSNHL, fluctuating sensorineural hearing loss; RV, recurrent vestibulopathy; MD, Ménière's disease, ISSNHL, idiopathic sudden sensorineural hearing loss; p, p-value; ^*^statistically significant difference (p < 0.05); HL, hearing loss.

**Figure 2:**
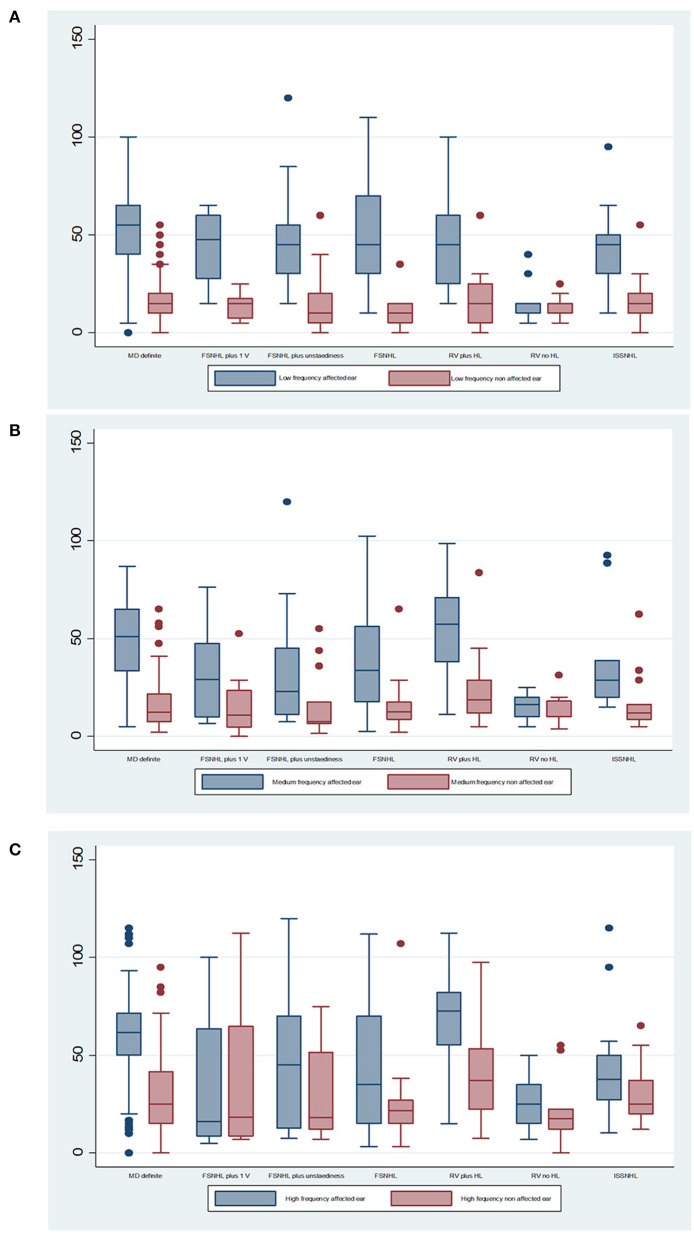
Mean audiometric thresholds for **(A)** low frequency (0.25 kHz), **(B)** mean pure tone average (PTA) for the 0.5, 1, 2, and 3 kHz, and **(C)** mean high frequency (4 and 6 kHz). The median is the middle line of the box plot, the bottom line represents the 25th percentile, and the top line of the diagram represents the 75th percentile. The points are outlier values that indicate that the value is more than 1.5 times the interquartile range above the 75th percentile.

Hearing loss in each group is shown in Figure 2 for each category of diagnosis: the results are shown for the affected and non-affected ear for each subgroup in the affected and non-affected ear, and for three categories: low frequency (0.25 kHz), mean pure tone average (PTA) for the 0.5, 1, 2, and 3 kHz and mean high frequency (4 and 6 kHz).

According to findings shown in Table 3, there are statistically significant differences between groups in all three frequency groups in the case of the affected ear, when comparing the percentage of patients with hearing loss lower or higher than 50 dB in each group. There are no statistically significant differences for the non-affected ear.

**Table 3:** Distribution of patients in each category of diagnosis for audiometric threshold below or higher or equal to 50 dB hearing loss.

		**FSNHL**			**RV**		**MD definite**	**ISSNHL**	***p***
		**No vestibular symptoms**	**Unsteadiness**	**Vertigo**	**No HL**	**Plus HL**			
Low frequency (0.25 kHz), affected ear (%)	<50 dB	53.3	53.3	50	100	52	30.1	53.3	0.02*
	≥50 dB	46.7	46.7	50	0	48	69.9	46.7	
Low frequency (0.25 kHz), non-affected ear (%)	<50 dB	100	93.3	100	100	96	97.6	92.9	0.84
	≥50 dB	0	6.7	0	0	4	2.4	7.1	
PTA, affected ear (%)	<50 dB	73.3	80	75	100	40	48.2	86.7	0.01*
	≥50 dB	26.7	20	25	0	60	51.8	13.3	
PTA, non-affected ear (%)	<50 dB	93.3	93.3	87.5	100	96	96.4	92.8	0.9
	≥50 dB	6.7	6.7	12.5	0	4	3.6	7.1	
High frequency (4 and 6 kHz), affected ear (%)	<50 dB	66.7	60	62.5	88.9	16	24.1	73.3	0.00*
	≥50 dB	33.3	40	37.5	11.1	84	75.9	26.7	
High frequency (4 and 6 kHz), non-affected ear (%)	<50 dB	93.3	73.3	62.5	77.8	72	79.5	85.7	0.6
	≥50 dB	6.7	26.7	37.5	22.2	28	20.5	14.3	

PTA, mean pure tone average for 0.5, 1, 2, and 3 kHz. FSNHL, fluctuating sensorineural hearing loss; RV, recurrent vestibulopathy; MD, Ménière's disease, ISSNHL, idiopathic sudden sensorineural hearing loss; p, p-value; ^*^statistically significant difference (p < 0.05); HL, hearing loss.

The results of vestibular examination are summarized in Table 4. As expected, there is high variability depending on the group. The rate of abnormal caloric testing is higher among RV without HL (80%) and definite MD (68.8%), but 25% of patients with FSNHL with no vestibular symptoms and 33.3% of ISSNHL showed abnormal results. The rate of abnormal vHIT in ISSNHL was also 40%. For the remaining groups, the rate of abnormal vHIT was 26.3% in the MD definite group, 25% for FSNHL plus unsteadiness, 22.2% for the FSNHL, 39.1% for the RV with HL, and 12.5% for the RV without HL group, and no abnormal vHIT for the group FSNHL with vertigo. The abnormal vHIT response for the horizontal semicircular canal of the affected ear was not specifically evaluated, but nevertheless, the discrepancies between the caloric test and vHIT (with an abnormal caloric test and a completely normal vHIT) is noteworthy in several groups. In the MD definite group, 68.8% of patients had an abnormal caloric test, but only 26.3% showed any kind of abnormality in vHIT. The rate of asymmetry in both oVEMPS and cVEMPS is higher in group FSNHL with vertigo and definite MD (33.9 and 31.6%: FSNHL with vertigo, 38.2 and 35.2%: definite MD, respectively).

**Table 4:** Summary of findings in the vestibular examination.

		**FSNHL**			**RV**		**MD DEFINITE**	**ISSNHL**
		**No vestibular symptoms**	**Unsteadiness**	**Vertigo**	**No HL**	**Plus HL**		
Caloric test (%)	Normal	75.0	33.3	50.0	20.0	44.4	31.3	66.7
	Abnormal	25.0	66.7	50.0	80.0	55.6	68.8	33.3
Spontaneous nystagmus (%)	Yes	8.3	21.4	87.5	11.1	26.1	37.8	14.3
	No	91.7	78.6	12.5	88.9	73.9	62.2	85.7
vHIT (%)	Normal	77.8	75.0	100.0	87.5	60.9	73.8	60.0
	Abnormal	22.2	25.0	0.0	12.5	39.1	26.3	40.0
oVEMP (mean, SD)		26.7 (11.1)	24.0 (25.2)	33.9 (17.9)	19.8 (16.9)	19.8 (16.9)	38.2 (26.9)	33.25 (23.3)
cVEMP (mean, SD)		27.4 (24.8)	27.3 (18.9)	31.6 (26.4)	15.1 (14.5)	30.8 (21.7)	35.2 (26.8)	13.0 (8.8)

*FSNHL, fluctuating sensorineural hearing loss; RV, recurrent vestibulopathy; MD, Ménière's disease, ISSNHL, idiopathic sudden sensorineural hearing loss; HL, hearing loss*.

### Magnetic Resonance Imaging Detection of Endolymphatic Hydrops

**Figure 3:**
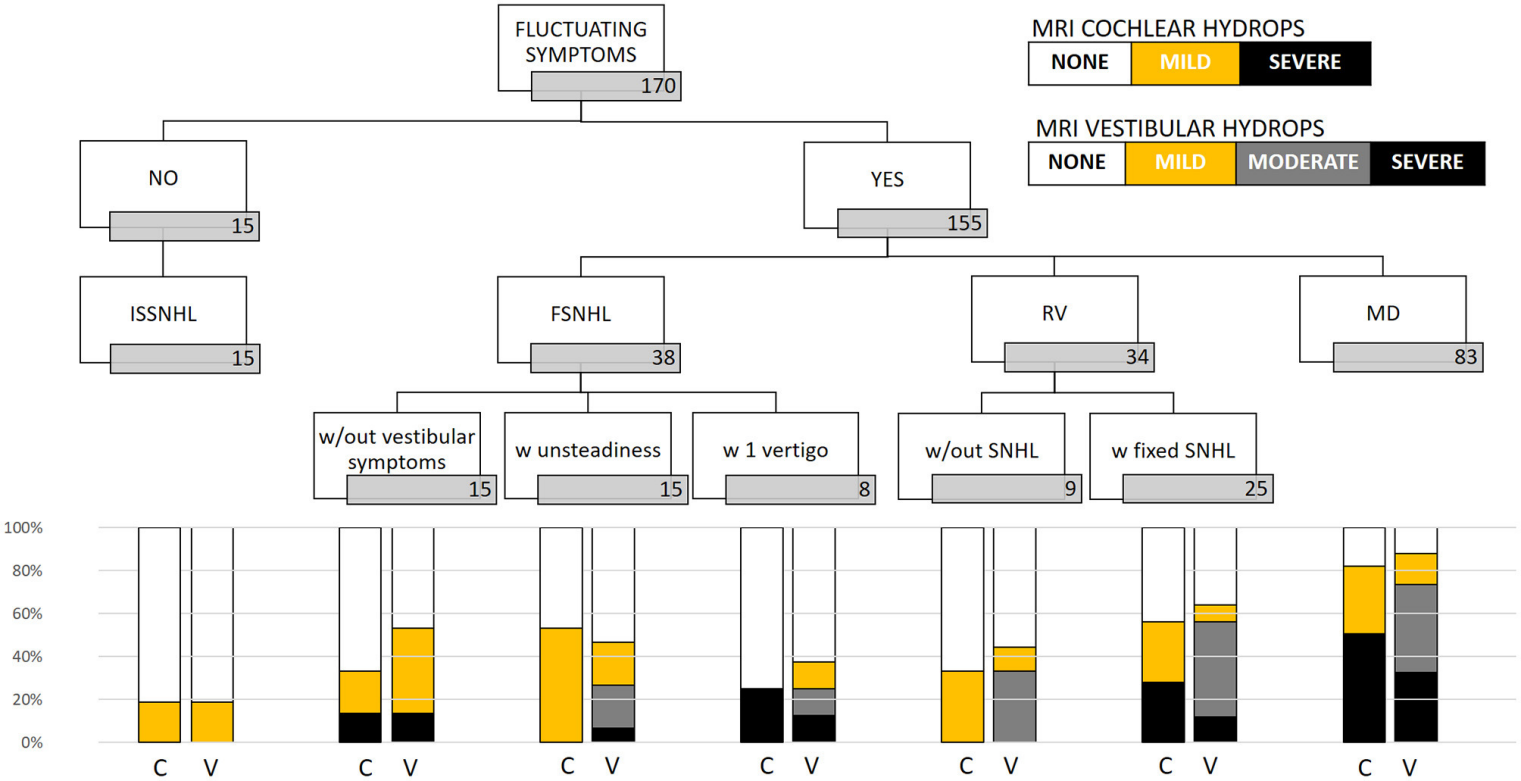
Distribution of MRI hydrops according to category of disease in the affected ear. In the cochlea, only none, mild, and severe results are given; in the vestibule, the four (none, mild, moderate, and severe) are given. C, cochlear hydrops; V, vestibular hydrops.

**Figure 4:**
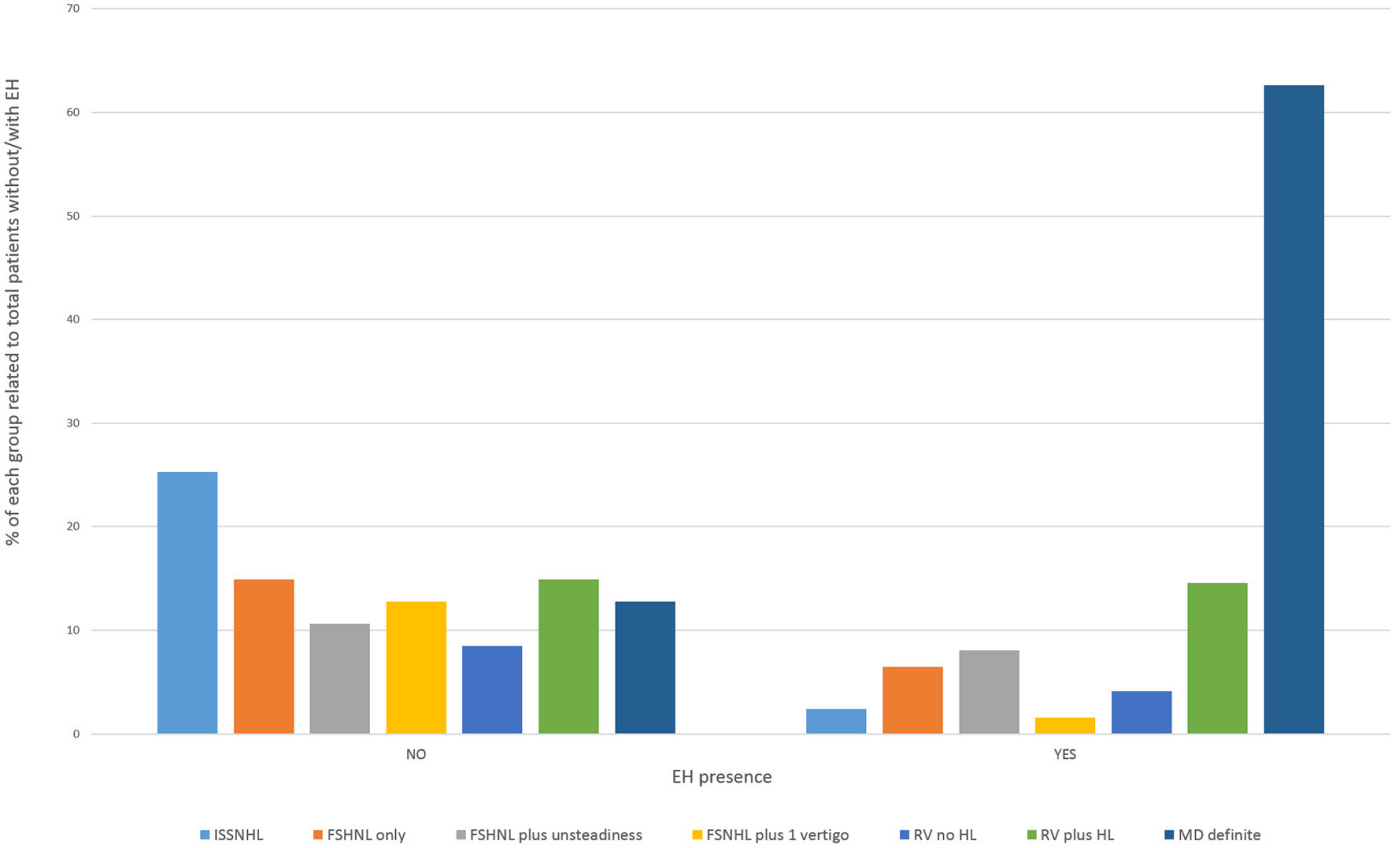
Cochlear or vestibular hydrops in the affected ear according to category of the disease.

In the clinically affected ear, cochlear hydrops was detected in 103 of 170 patients (60.6%); it was mild in 50 (29.4%) and severe in 53 (31.2%) ears. Vestibular hydrops was detected in 114 patients (67.1%), and was mild in 28 (16.5%), moderate in 52 (30.5%), and severe in 34 (20%). The distribution of findings is shown in Table 5 and Figure 3. Both cochlear and vestibular hydrops were detected in 94 (55%) patients in the affected ear. In Figure 4, the contribution (percentage) of each group to the patients without EH vs. the patients with EH is shown, and major differences are observed between groups. No EH either at the cochlea or at the vestibule was detected in 18 (47%) of the patients with FSNHL and in 11 (32%) of the patients with RV in the affected ear. In those two groups, seven (20%) and six (16%) patients, respectively, were found to have severe cochlear and/or severe vestibular EH.

**Table 5:** Distribution of the degree of hydrops in the affected and unaffected ear by diagnosis in number of patients.

	**ISSNHL**		**FSNHL**		**RV**		**MD**
	**ISSNHL**	**No vestibular**	**Unsteadiness**	**1 vertigo**	**No SNHL**	**Fixed SNHL**	**Definite**
**AFF Cochlea**							
No	12	10	7	6	6	11	15
Mild	3	3	8	0	3	7	26
Severe	0	2	0	2	0	7	42
**AFF Vestibule**							
None	12	7	8	5	5	9	10
Mild	3	6	3	1	1	2	12
Moderate	0	0	3	1	3	11	34
Severe	0	2	1	1	0	3	27
**Non-AFF Cochlea**							
No	11	15	12	8	7	24	72
Mild	4	0	3	0	2	1	10
Severe	0	0	0	0	0	0	1
**Non-AFF Vestibule**							
None	14	15	13	8	6	20	62
Mild	1	0	1	0	2	5	14
Moderate	0	0	1	0	1	0	6
Severe	0	0	0	0	0	0	1

*FSNHL, fluctuating sensorineural hearing loss; RV, recurrent vestibulopathy; MD, Ménière's disease, ISSNHL, idiopathic sudden sensorineural hearing loss; SNHL, sensorineural hearing loss; AFF, affected ear; NON-AFF, non-affected ear*.

Perilymphatic enhancement was observed only in the affected ear. It was detected only in the cochlea of 30 (17.6%) patients, only in the vestibule in 11 (6.5%), and in both cochlea and vestibule in 16 (9.4%). Perilymphatic enhancement was found in 13 of 46 (28.2%) ears without either cochlear or vestibular EH. Of these, three were definite MD, three were FSNHL plus unsteadiness, two were FSNHL with one vertigo spell, two were FSNHL without vestibular symptoms, two were recurrent vertigo with hearing loss, and one was ISSNHL.

In the non-affected ear, cochlear hydrops was detected in 21 (12.4%) patients; it was mild in 20 and severe in one case. Vestibular hydrops was detected in 32 (18.8%) patients and was mild in 23 (13.5%), moderate in eight (4.7%), and severe in one case (0.6%). Only in four ears were both cochlear and vestibular hydrops detected.

We observed an association between the group of patients with definite diagnosis of MD and hydrops development risk compared with patients with diagnosis of atypical MD or the ISSNHL group. Patients with definite diagnosis of MD have a significant increase in risk of hydrops in MRI compared with the atypical MD + ISSNHL group [OR 11.43 (4.5–29.02); *p* < 0.001]. After adjusting for traditional cardiovascular risk factors, we found similar results (Table 6). Once the reference category was changed, taking the ISSNHL group as the reference, we observed that the group with definite diagnosis of MD had a statistically significant increase in risk of hydrops [OR 55.2 (11.9–253.9); *p* < 0.001]. To a lesser extent, we also observed a significant increase in risk for the RV group [OR 9.9 (2.1–38.9); *p* = 0.003] and for the FSNHL group [OR 5.1 (1.2–21.7); *p* = 0.03], both compared with the reference group. After adjusting for traditional cardiovascular risk factors, we found similar results in all groups (Table 7).

**Table 6:** Odds ratio and 95% confidence intervals of hydrops according to Ménière's disease.

	**MD “atypical” and ISSNHL**	**MD definite**	***p***
N	87	83	
Adjusted for age and sex	1 (reference)	11.3 (4.4–28.8)	<0.001
Multivariable adjusted model^*^	1 (reference)	13.6 (4.9–37.9)	<0.001

* *Multivariable adjusted: adjusted for age, sex, and vascular risk factors (hypertension, diabetes, and dyslipidaemia)*.

**Table 7:** Odds ratio and 95% confidence intervals of hydrops according to ISSNHL as the reference group.

	**ISSNHL**	**MD definite**	***P***	**FSNHL**	***p***	**RV**	***p***
N	15	83		38		34	
Adjusted for age and sex	1 (reference)	55.2 (11.9–253.9)	<0.001	5.1 (1.2–21.7)	0.03	9.9 (2.1–38.9)	0.003
Multivariable adjusted model^*^	1 (reference)	97.7 (15.6–611.4)	<0.001	6.9 (1.2–39.2)	0.03	14.9 (2.5–87.5)	0.003

* *Multivariable adjusted: adjusted for age, sex, and vascular risk factors (hypertension, diabetes, and dyslipidemia)*.

## Discussion

The pathological hallmark of MD is underlying EH, which was reported for the first time in 1938 (30, 31). As stated in the *Introduction* section, recent developments of high-resolution MR imaging of the inner ear have now enabled us to visualize *in vivo* EH in patients with clinical MD (32). The existing knowledge in this field supports the idea that not only is EH responsible for MD but also for other clinical malaises not fulfilling definite MD criteria (33). Previous authors (34) have also shown that, while clinical symptoms fluctuate in definite MD patients, EH is quite stable and tends to progress in the long term. We have shown in this paper that EH, as detected by MRI, is a major finding in patients with any type of inner ear disorder in which auditory or vestibular symptoms fluctuate, and its presence could, hence, imply an increased risk of evolution to definite MD in those patients.

For patients who met the criteria of definite MD (both in the AAOHNS 1995 and Barany 2015 classifications), the rate of EH is 96% in our group. These results agree with previous studies (11). The risk of EH compared with the other groups is high, especially when taking as a reference ISSNHL [OR 55.2 (11.9–253.9); *p* < 0.001]. These patients could well be screened before in particular with electrophysiological methods (frequency tuning of the VEMP), which has shown very good correlation to EH detection (21, 35).

In the case of “probable,” “possible,” and “atypical” MD, the norm is misunderstood. We have shown here (1) that the risk of EH among patients with definite MD is higher than in atypical MD or ISSNHL [OR 11.43 (4.5–29.2); *p* < 0.001], suggesting that the more the inner ear is clinically affected, the higher the risk an underlying EH is present and (2) that the risk of EH in the “atypical” group is lower than in the definite MD group but remains significant, taking the ISSNHL as reference. Given these findings, we believe there is an argument to promote a more detailed classification under the MD spectrum or “hydropic inner ear disease” (1). This is also based on the detailed analysis of the findings as we observe that the MRI-detected EH is more severe in cases in which the clinical presentation includes vertigo and hearing loss as shown in Figure 3. The presence of “severe EH” is only depicted in cases of FSNHL with vertigo (25% cochlear and 12.5% vestibular), in RV with SNHL (28% cochlear and 12% vestibular), and in definite MD (51% cochlear and 32.5% vestibular).

Over the last few years, the presence of EH in inner ear disease entities not fulfilling the clinical criteria for definite MD appears to have increased. In patients with FSNHL, previous authors demonstrated a prevalence of EH of 82.5%, being more prevalent in the vestibule than in the cochlea (36). We found cochlear EH in 39% of patients with FSNHL and vestibular EH in 47.3%. In those with RV, the number of patients with cochlear EH was 50% and with vestibular hydrops 58.8%, a very similar number to that mentioned by previous authors (37). These are patients in whom EH detection by MRI could be relevant for treatment and follow-up. From previous work, we know that the prognosis in those patients is very good (15) as the symptoms disappear, or the vertigo is resolved in 66% over a 12- to 62-month time period (38). In that work, neither age, sex, disease duration, frequency of attacks, nor time since first attack showed significant differences between the group that became inactive in the long term and those who were still active or did develop MD. The only significant difference was that, in the latter, the caloric test was much more frequently abnormal than in the former. It will be interesting to see how these patients evolve in future work.

All these data support the idea of maintaining the cochlear and vestibular categories as subtypes of MD or, at least, of introducing the location and quantity of hydrops in those cases. The utriculo-endolymphatic valve may play a role in maintaining the independence of the superior part (utricle and canals) from the inferior part (saccule and cochlear duct) (39) and be responsible of the cochlear and vestibular subtypes of MD.

Consequently, EH identification in patients with fluctuant hearing loss and recurrent vertigo cannot be dismissed in their diagnostic process. We agree that this technique should not be considered the gold standard for the diagnosis of definite MD (40) as now considered. In particular, this is observed in some cases where, after detailed medical evaluation, there is no identification of EH in the MRI as shown in our work and by others who mention that up to 10–33% of patients who fulfill the criteria for definite MD do not have MRI-demonstrable changes of hydrops (41, 42). This clinical radiologic discrepancy still reflects an incomplete understanding of the disease process and the need for additional imaging biomarkers of disease activity in MD beyond EH. It could be due to differences in phenotypes that have been defined in unilateral (4) and bilateral (5) definite MD. Additionally, taking “perilymphatic enhancement” into consideration as a marker for MD could provide a more robust diagnostic criterion (29); in our work, 13 out of 43 symptomatic ears without EH in the “atypical MD group” showed perilymphatic enhancement as the only MRI finding and, thus, could indicate added diagnostic value.

In ISSNHL, mild EH was observed in 20% of the cases. To the best of our knowledge, the evidence of EH within this clinical entity is absent (43) or very low (44). In the latter case, it was described in 2/8 of patients (one cochlear EH and the other cochlear and vestibular EH), in both cases being considered as a secondary EH. In our study, the mean time since sudden hearing loss to MRI study is always under 30 days; therefore, we cannot explain such findings as a secondary EH. Also, the rate of EH is higher in our study. This finding may be explained by two facts. First, EH is found in both affected and unaffected ears. A recent study shows how the relation of endolymphatic volume to total fluid space in the inner ear is significantly different between normal controls and of patients with ISSNHL when the cochlea of both the affected and unaffected ears were analyzed (45). Second, the imaging technique used may favor this finding. Some degree of hydrops in normal subjects is expected as slight apical cochlear EH has been described in 15% of normal subjects at the oto-pathological record (46). In our work, we used the REAL-IR MRI technique, which allows direct discrimination of bone (gray signal), perilymph (white signal), and endolymph (black signal), with robust and easy EH evaluation. Because of this, the REAL-IR sequence seems to be superior to FLAIR (in which both endolymph and bone appear black), improving slight EH detection in the cochlea where the separation between the endolymph of the scala media and the bone is minimal (47). A similar finding of slight EH has not been mentioned in normal people for the vestibule; nevertheless, should the three-grade scale for vestibular EH have been used instead of the four-grade scale, those cases with mild vestibular EH would have been considered normal. This suggests that the four-grade scale for vestibular EH could be more sensitive but less specific than the three-grade scale. We may, thus, conclude that significant EH presence in ISSNHL is still anecdotic. The continuum for sudden hearing loss has been well-evaluated in a recent nationwide survey in Japan (48) revealing that the incidence of ISSNHL was 60.9 per 100,000 population. A follow-up and in-depth study of this population may shed some light on how this malaise may evolve, including EH. As a corollary of the previous findings, we stress our interest in not incorporating normal subjects into this study as the qualitative measurement we use probably could not be able to discriminate mild cochlear EH as do quantitative newer methods (49).

That EH MRI may play a role in the diagnosis of inner ear disease is a real concept only limited by technological accessibility, in particular, when trying to anticipate irreversible cochlear and vestibular findings, is of interest in cases of hearing loss and vertigo that may evolve into a definite MD. Our findings indicate that the expected number of patients who probably will have “definite” MD after follow-up coincides with those in whom we found both severe cochlear and severe vestibular EH. This was two (5.2%) in the case of FSNHL and three (8.8%) in the case of RV. Those patients began close follow-up and are currently being treated with diuretics. In a similar way, it has been recently shown that, in patients with ISSNHL, an increase in the endolymphatic space could render them prone to developing FSNHL (45). Also, there is a substantial number of patients with cochlear MD who will proceed to definite MD, and previous work has shown that this may occur in almost 80% of cases (50).

At this point, there may be some confusion regarding the different ways of classifying the severity of EH in MRI, and there is urgent need for agreement on a common methodology. Regarding imaging, in the case of cochlear EH, a two-grade (absent or present) or a three-grade (absent, mild, or severe) classification system is generally used. In the case of vestibular EH, a three-grade (absent, mild, or severe) classification system has been proposed by some authors (25, 42) while a four-grade scale (absent, mild, moderate, or severe) has also been used (26, 29, 51). Unfortunately, it is still not possible to evaluate EH of the semicircular canals due to its very small size and lack of spatial resolution. Occasionally, increased asymmetric hyperintensity of the perilymph is additionally found both in cochlear and vestibular compartments. This finding is interpreted as an increased diffusion of contrast material to the perilymph due to altered permeability of the blood–labyrinth barrier, presumably secondary to active inflammation (28, 29). In addition, herniation of vestibular EH toward the non-ampullar end of the horizontal semicircular canal (HSC) has been described both in histopathology and EH MRI (27). The relevance of all these MRI findings, both in themselves and in combination, requires standardization.

Regarding the vestibular tests, a discrepancy between the caloric test and vHIT was noteworthy in some groups, especially MD, as previously published (26, 52). Analysis of this dissociated response (with an altered caloric test but normal vHIT results for the HSC of the affected ear despite analyzing the function of the same vestibular organ) was not the primary goal of this study, and specific alteration of the horizontal semicircular canal was not recorded for comparison with a caloric test (only global results of normal or abnormal vHIT), and so it was not further analyzed. Nevertheless, the discrepancy between the caloric test result and vHIT has even been proposed as a marker of MD. With the results of this work, we must now consider that it may be much more common than expected also in other groups and probably be a closer relationship to hydrops than to MD itself. Also, alternative analysis of audiovestibular tests as the total number of involved vestibular end organs as done recently (9) is a promising means for evaluation of the severity of MD, and possible correlation with EH in MD and other groups should be explored in a prospective way.

As a limitation of the study, we must mention that EH was evaluated according to semiquantitative visual scales. Although volumetric evaluation of EH has been reported (53), it is not normally used in routine clinical practice, mainly because there is no dedicated software that has been developed. However, recent advances in the use of artificial intelligence with deep learning (still to be circulated) represent a major step forward for the purpose of providing an objective measure (54). Nonetheless, the correlation obtained between semiquantitative scales and volumetry is very good (55).

In this study, clinical features such as age, sex, side, disease duration, migraine, and vascular risk factors have a similar distribution among different clinical entities. Thus, patients included are homogeneous, minimizing the risk of bias in the interpretation of results so we do not consider this to be a relevant limitation.

Another limitation is that the categories were taken from an old reference, and a proper “vestibular migraine” category was not included. Of those in the group “Atypical MD Vestibular,” five of 34 could have been classified as definite vestibular migraine, after reviewing the records. Only two, one in each of the categories here used, showed vestibular hydrops, which in both cases was “moderate” (56).

The transversal nature of the study also limits its conclusions, asking for longitudinal prospective studies evaluating the clinical and radiological evolution of these patients and the possible association of MRI EH in non-definite MD patients and posterior transformation to definite MD.

Also, potential correlations between audiovestibular tests and the localization of EH or with the presence of EH herniation in the semicircular canals were not analyzed and could be evaluated in future works.

## Conclusion

MRI EH is found in a percentage of patients with fluctuating audiovestibular symptoms not fulfilling the actual diagnostic criteria for definite MD. This percentage is variable depending on the audiovestibular symptoms, from a low percentage of only slight (and even questionable) EH in ISSNHL to a moderate percentage in patients with FSNHL with one vertigo crisis or recurrent vertigo with fixed SNHL. The percentages of severe cochleovestibular EH are similar to the current reported percentages of progression to definite MD in those groups, suggesting that presence of EH by MRI could be related to the risk of progression to definite MD, and advising longitudinal follow-up studies already under way. MR EH imaging in these patients is, thus, recommended.

## Data Availability Statement

The datasets presented in this article are not readily available because they have not been uploaded to a repository, but as previously stated questions related to the accuracy or integrity of any part of the work will be appropriately investigated and resolved. Requests to access the datasets should be directed to Nicolás Pérez-Fernández, nperezfer@unav.es.

## Ethics Statement

Ethical review and approval was not required for the study on human participants in accordance with the local legislation and institutional requirements. Written informed consent for participation was not required for this study in accordance with the national legislation and the institutional requirements.

## Author Contributions

All authors made substantial contributions to the conception and design of the work, data acquisition, analysis and/or interpretation, work draft, and critical review. All authors approved the final version to be published. All authors agreed to be accountable for the content of the work in ensuring that questions related to the accuracy or integrity of any part of the work are appropriately investigated and resolved.

## Conflict of Interest

The authors declare that the research was conducted in the absence of any commercial or financial relationships that could be construed as a potential conflict of interest.
